# Supplementary material for: Genome evolution in an ancient bacteria-ant symbiosis: parallel gene loss among Blochmannia spanning the origin of the ant tribe Camponotini
Source: PeerJ. 2015 Apr 2;3:e881. doi: 10.7717/peerj.881 (PMC4389277; doi:10.7717/peerj.881)
Supplement: Table S2 [file peerj-03-881-s002.doc]

**Supplemental Table S2. BLASTN analysis of contigs from *de novo* assemblies.**

| Read dataset | Contig # | Length of contig (bp) | BLASTN top hit | | | | |
| --- | --- | --- | --- | --- | --- | --- | --- |
|  |  |  | Name | Accession # | Evalue | % coverage | % id |
| *C. obliquus* | 6 | 1373 | *Wolbachia* | CP001391 | 0 | 100 | 83 |
|  | 18 | 861 | *Wolbachia* | CP003883 | 0 | 99 | 94 |
|  | 51 | 2105 | *Solenopsis* *invicta* mito | HQ215538 | 0 | 98 | 71 |
|  | 167 | 1305 | *Wolbachia* | AE017196 | 0 | 99 | 91 |
|  | 171 | 1278 | *Wolbachia* | HM209308 | 0 | 99 | 99 |
|  | 175 | 704 | *Wolbachia* | CP001391 | 5e-160 | 91 | 76 |
|  | 202 | 2272 | *Leptothorax* *acervorum* 18S rRNA gene | X89492 | 0 | 84 | 99 |
|  | 236 | 1461 | *Wolbachia* | JX987274 | 0 | 98 | 82 |
|  | 240 | 1190 | *Wolbachia* | CP003884 | 0 | 100 | 94 |
|  | 311 | 1610 | *Wolbachia* | CP001391 | 0 | 99 | 81 |
|  | 334 | 1994 | *Wolbachia* | AM999887 | 0 | 64 | 93 |
|  | 401 | 1197 | *Wolbachia* | JX987269 | 0 | 99 | 94 |
|  | 402 | 751 | *Camponotus* sp. AEAO 28S rRNA gene | JN134385 | 0 | 71 | 99 |
|  | 443 | 813 | *Camponotus* sp. BCA01 28S rRNA gene | EF012974 | 0 | 100 | 99 |
|  | 518 | 780 | *Wolbachia* | CP003883 | 0 | 99 | 84 |
| *P. turneri* | 224 | 2704 | *Leptothorax* *acervorum* 18S rRNA gene | X89492 | 0 | 70 | 99 |
|  | 273 | 1027 | *Camponotus* *chromaiodes* mito | JX966368 | 0 | 99 | 84 |
|  | 547 | 1116 | *Camponotus* sp. AEAO 28S rRNA gene | JN134385 | 0 | 44 | 99 |
|  | 1132 | 809 | *Polyrhachis* sp. 28S rRNA gene | EF013045 | 0 | 100 | 100 |
|  | 1951 | 985 | *Chrysis* *splendidula* mito | HM071081 | 2e-155 | 99 | 74 |
|  | 2557 | 662 | *Formica* *sanguinea* mito | AM910841 | 2e-120 | 77 | 78 |
|  | 4137 | 1256 | *Vespula* *pensylvanica* 28S rRNA gene | AY859612 | 0 | 90 | 96 |
|  | 6523 | 507 | *Blochmannia pennsylvanicus* | CP000016 | 2e-41 | 65 | 72 |
